# Using an affinity analysis to identify phytoplankton associations

**DOI:** 10.1002/ece3.9047

**Published:** 2022-07-05

**Authors:** Weiju Zhu, Zhaojian Ding, Yangdong Pan, Quanxi Wang

**Affiliations:** ^1^ School of Science Qiongtai Normal Universtiy Haikou China; ^2^ Tropical Biodiversity and Bioresource Utilization Laboratory Qiongtai Normal University Haikou China; ^3^ Environmental Science and Management Portland State University Portland Oregon USA; ^4^ College of Life and Environmental Sciences Shanghai Normal University Shanghai China

**Keywords:** affinity analysis, Dishui Lake, functional groups, Huaihe River basin, phytoplankton association

## Abstract

Phytoplankton functional traits can represent particular environmental conditions in complex aquatic ecosystems. Categorizing phytoplankton species into functional groups is challenging and time‐consuming, and requires high‐level expertise in species autecology. In this study, we introduced an affinity analysis to aid the identification of candidate associations of phytoplankton from two data sets comprised of phytoplankton and environmental information. In the Huaihe River Basin with a drainage area of 270,000 km^2^ in China, samples were collected from 217 selected sites during the low‐water period in May 2013; monthly samples were collected during 2006–2011 in a man‐made pond, Dishui Lake. Our results indicated that the affinity analysis can be used to define some meaningful functional groups. The identified phytoplankton associations reflect the ecological preferences of phytoplankton in terms of light and nutrient acquisition. Advantages and disadvantages of applying the affinity analysis to identify phytoplankton associations are discussed with perspectives on their utility in ecological assessment.

## Graphical abstract

Our results indicated that the affinity analysis can be used to define some meaningful functional groups. The identified phytoplankton associations reflect the ecological preferences of phytoplankton in terms of light and nutrients acquisition. Advantages and disadvantages of applying the affinity analysis to identify phytoplankton associations are discussed with perspectives of their utility in ecological assessment.

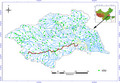

## 1 INTRODUCTION

Ecologists are facing challenges to decipher a rich amount of biological and environmental information embedded in an ecological community. The classification of a set of taxonomic units into functional groups based on morphology and species traits has been widely used in ecological research (Litchman & Klausmeier, 2008; Usseglio‐Polatera et al., 2000). If species are pooled into the same group based on similar morphological or physiological characteristics and developing ecological groups, that can help ecologists to better understand the interactions between biological communities and their environment. For example, stream macroinvertebrates have been categorized into functional feeding groups, such as scrapers, shredders, collector‐gatherers, collector‐filterers, and predators. Logez et al. (2013) suggested that similar fish assemblage functional structures will be found in similar environmental conditions.

Phytoplankton is one of the primary producers in aquatic ecosystems, their community structure can be a rapid and sensitive response to varying environmental conditions in aquatic systems and are therefore commonly used as excellent bio‐indicators (Reynolds, 2006). However, phytoplankton has a wide variety which brings some challenges for accurate identification to the beginner. Categorizing phytoplankton by their traits and functions was attempted a few decades ago. Reynolds et al. (2002) set a precedent in the classification of phytoplankton functional groups. Salmaso and Padisák (2007) developed the Morpho‐Functional Groups based on the phytoplankton's morphological and functional characteristics, such as body size, mobility, nutrient requirements, and other features. Kruk et al. (2010) applied morphology‐based functional groups (MBFG) approach to cluster phytoplankton organisms, and seven groups were defined according to the main morphological traits of phytoplankton such as cell volume, presence of flagella, and the ratio of surface area and volume. However, there are still some challenges for phytoplankton ecologists to apply functional concepts in phytoplankton research. For example, phytoplankton communities can be extremely rich (Reynolds, 2006) but may differ from region to region due to ecological factors. Classifying species into different functional groups requires an extensive amount of knowledge of the autecology of each species, and such information may not be readily available in the literature. Physiological data are not available for all phytoplankton species (Weithoff, 2003), which limits our abilities for developing a priori functional classification. On the other hand, environmental assessment of lakes using phytoplankton is urgently needed by water quality managers, especially in some rapidly developing regions such as China because of serious water pollution. A great deal of phytoplankton ecological studies have been conducted in Europe (EC Parliament and Council, 2000), North America (Arhonditsis et al., 2004), and China (Deng et al., 2014). The implementation of water programs has generated an enormous amount of phytoplankton data with large spatial scales using standardized field protocols. How to effectively use these “data sets” to enhance our current understanding of phytoplankton assemblages in relation to their environments and water resource management still remains challenging.

In order to provide a better understanding of the ecological information of phytoplankton associations, we introduce an affinity analysis called association rule for identifying phytoplankton associations. Association rule is a machine‐learning method for discovering co‐occurrence relationships among activities performed by specific individuals or groups in a large database using simple statistical performance measures. There have been many successful business applications for applying the method in finance, telecommunication, marketing, retailing, and web analysis (Chen et al., 2005). In ecological studies, we assume that each sampling site or sampling date is a “transaction” in a business setting and each species is an item and then develop the associations. Many researches focus on phytoplankton spatial and temporal variations in lakes and rivers, and therefore this study identified phytoplankton associations from spatially and temporally data sets, respectively. The main objective of this study was to use affinity analysis to aid identification of the candidate associations of phytoplankton and then assess the relationships between the candidate phytoplankton associations and environmental factors using the redundancy analysis (RDA).

## 2 METHODS

### 2.1 Data preparation

#### 2.1.1 River phytoplankton: A spatial data set

**FIGURE 1:**
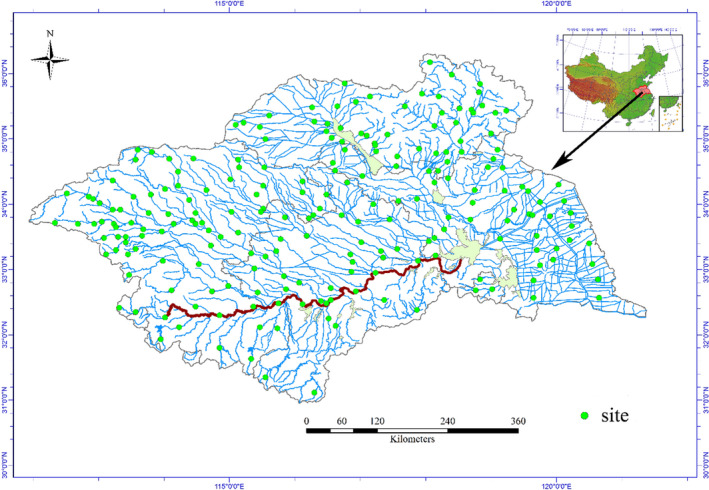
The map of China showing the location of HRB and the HRB showing all sampling stations of phytoplankton

River phytoplankton data were collected as a part of the Water Pollution Control Program in the Huaihe River Basin (30°55′–36°36′N, 111°55′–121°25′E) (HRB). The basin, with a drainage area of 270,000 km^2^, is located between the Yangtze River and the Yellow River in China (Wang & Xia, 2010). It forms a geographical separation between northern and southern China. Phytoplankton samples were collected from 217 randomly selected sites during the low‐water period from May 1 to May 31 in 2013 (Figure 1). Detailed field and lab methods can be found in Zhu et al. (2015). At each site, three cross‐section transects were established. We measured in situ environmental variables including water temperature (WT), pH, conductivity (Cond), turbidity, and total suspended solids (TSS) using a portable HACHCDC40105. We also measured Secchi depth (SD), water depth, stream width, water velocity, and elevation. Spectrophotometer (DR5000) was used to measure total phosphorus (TP), total nitrogen (TN), and chemical oxygen demand (COD_Mn_) according to standard methods (NEPAC, 2002).

For phytoplankton, a 1 L sample from the 0.5 m depth below surface was collected from three cross‐section transects, respectively. After complete mixing, a 1 L sample was preserved with 1% Lugol's iodine solution immediately in the field and concentrated to 50 ml after sedimentation for 48 h. After complete mixing, 0.1 ml of the concentrated sample was counted directly in a 0.1 ml counting chamber under a microscope at 400× magnification. Phytoplankton was identified according to the reference book by Hu and Wei (2006). At least 400 algal units were counted in each sample. Phytoplankton biomass was expressed as wet biomass and was estimated for individual species by assigning a geometric shape similar to the shape of each phytoplankton species (Hillebrand, 1999).

#### 2.1.2 Lake phytoplankton: A temporal data set

**FIGURE 2:**
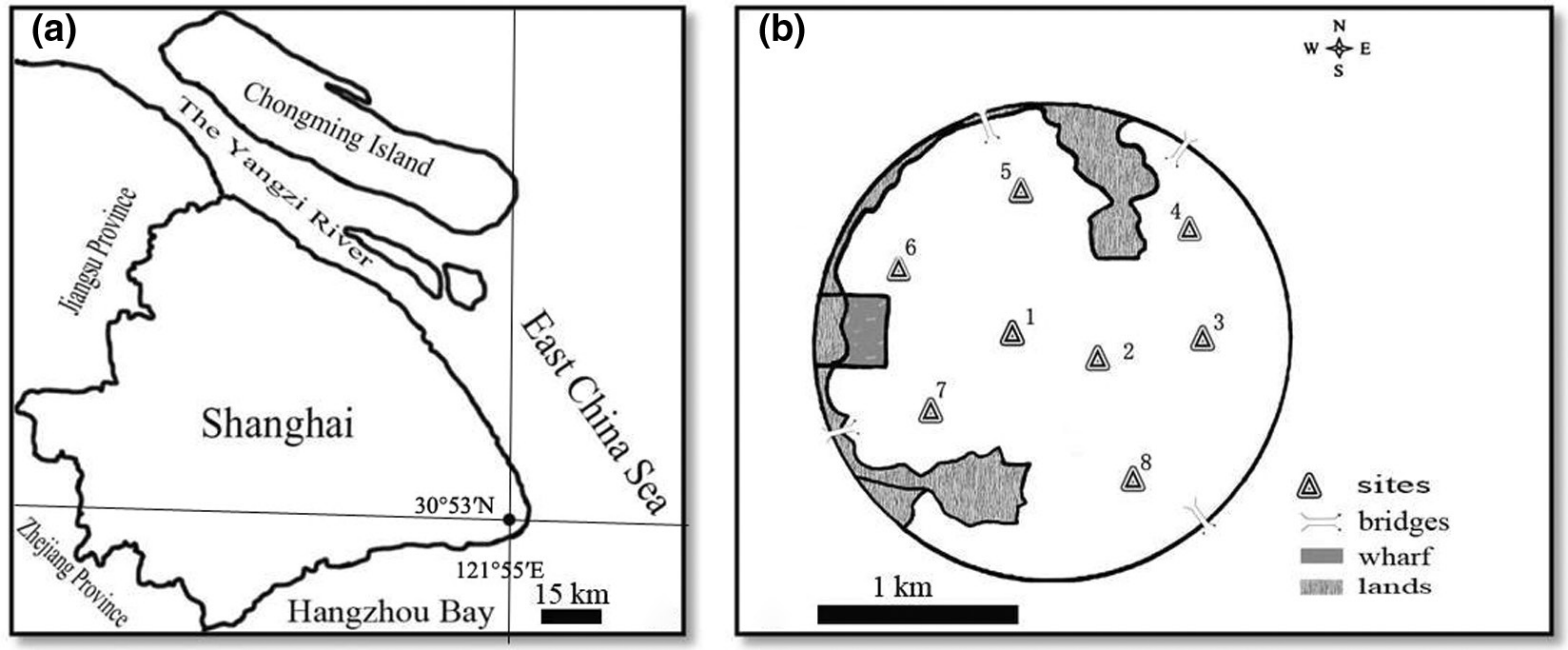
The map of Shanghai (a) and Dishui Lake (b) showing all sampling stations of phytoplankton

Monthly phytoplankton samples were collected during 2006–2011 to assess the temporal variability of phytoplankton assemblages in a man‐made pond. Dishui Lake (30°53′N, 121°55′E) is located in Pudong New Area District, southeastern Shanghai, China (Figure 2a). Detailed field and lab methods can be found in Zhu et al. (2013). Eight sampling stations were selected in the lake (Figure 2b). We measured in situ environmental variables including water temperature (WT), pH, conductivity (Cond), turbidity, total suspended solids (TSS), and Secchi depth (SD). Total phosphorus (TP), total nitrogen (TN), and chemical oxygen demand (COD_Mn_) were measured according to standard methods.

### 2.2 Data analysis

We selected 38 species in HRB and 23 species in Dishui Lake, respectively, after excluding “rare” taxa from analyses. The rare taxa were defined as those with average relative biomass (RB) <0.5% and occurred at <10 sites/samples (Zhu et al., 2015).

### 2.3 Data format

The data set was formatted as an M × N matrix, where the row represents different sampling sites S1, S2, S3…, Sn and the column represents phytoplankton species G1, G2, G3…Gn. Each element [i,j] represents the occurrence of the species j in the sample i (Table 1).

**TABLE 1:** The data format for identifying phytoplankton associations

Sites/species	G1	G2	G3	G4	G5	G6	……
S1	1	0	1	1	0	0	
S2	0	1	0	0	1	0	
S3	1	1	1	0	0	0	
S4	0	0	1	0	1	1	
S5	0	0	0	1	0	1	
S6	0	1	1	0	1	0	
S7	1	0	0	1	1	1	
S8	1	0	0	1	0	1	
S9	0	1	0	0	1	1	
S10	0	1	1	0	1	0	
S11	0	0	1	0	0	1	

### 2.4 Association rule

The matrix usually contains large amounts of data; therefore, data mining techniques are used to extract useful knowledge. We followed the association rule proposed by Agrawal et al. (1993).

Association rule is intended to capture a certain type of dependence among species represented in the database. The rule is defined as an implication of the form G1‐>G2, for example, an association rule between species in the form of G1‐>G 2 which means species 1 is also very likely to be observed with species 2 to form an association {G1, G2}.

The significance of the association rule is measured via support and confidence. The support of rule G1‐>G2 is the percentage of G1 and G2 occurring together. Confidence of rule G1‐>G2 is merely an estimate of the conditional probability of G2 given G1. If the confidence of rule G1‐>G2 is 1 that means G1 occurs in a particular site then G2 should occur in that site, too.

First, the binary phytoplankton data for identifying phytoplankton associations were constructed (Table 1), “S” represents the sampling site or time series, and “G” represents algae species. Secondly, the support of phytoplankton association was calculated. For instance, the association {G1, G3} has 18% support because the species G1 and G3 occur together in 2 of the 11 sites (Table 2). Finally, we calculated the confidence of each phytoplankton association (Table 3). For example, the confidence of the association {G1, G3} is 0.5 because species 3 occurs at half of times that also contains species 1.

**TABLE 2:** Calculate the frequency of associations

Phytoplankton associations	Site	Number of sites	Support (%)
{G1,G3}	S1,S3	2	18
{G1,G2}	S3	1	9
{G3,G5}	S4,S6,S10	3	27
{G1,G2,G3}	S3	1	9
{G2,G3,G5}	S6,S10	2	18
{G1,G4,G5,G6}	S7	1	9

**TABLE 3:** Calculate the confidence of associations

Phytoplankton associations (X and Y)	Number of sites (X)	Number of sites (both X and Y)	Confidence (both X and Y/X)
{G1} and {G3}	4	2	0.5
{G1} and {G2}	4	1	0.25
{G3} and {G5}	6	3	0.5
{G1,G2} and {G3}	1	1	1
{G2} and {G3,G5}	5	2	0.4
{G1,G4} and {G5,G6}	3	1	0.33

We identified the phytoplankton associations based on both support >=50% and confidence >= 0.8 (Wang et al., 2022).

All analyses were performed using R software (R Development Core Team, 2013). Specifically, we used the R package “arules” for the affinity analysis, “vegan” for detrended correspondence analysis (DCA), and redundancy analysis (RDA) and “packfor” for forward selection analysis (Dray et al., 2013; Hahsler et al., 2014; Oksanen et al., 2013).

The environmental factors except pH value are transformed to the form of log(*x* + 1), and the data of phytoplankton species are transformed by the Hellinger method. The environmental factors are selected by forward selection in RDA, in which the *p* value is less than .05 by the Monte Carlo permutation test.

## 3 RESULTS

Almost one‐third of taxa (28.95%) occurred at 50% of all sites or more in the HRB (Table 4). Five species (*Cryptomonas erosa*, *Chroomonas acuta*, *Cyclotella meneghiniana*, *Scenedesmus quadricauda*, *Navicula cryptocephala*) occurred at more than 70% of the sites. Two Euglenophytes, two Chrysophytes and *Pandorina morum* occurred at less than 10% sites. Eighteen of 23 taxa were present in Dishui Lake during more than half of the sample dates (Table 4). Only five taxa had less than 50% occurrence (e.g., *Carteria* sp., *Amiphiprora* sp., *Cryptomonas ovata*, *Cyclotella* sp., *Melosira varians*).

**TABLE 4:** The summary of phytoplankton taxa frequency in HRB and Dishui Lake

Taxa	Frequency (%)
Huaihe River Basin (*n* = 217)	
*Cryptomonas erosa* (*C*.*erosa*)	88
*Cyclotella meneghiniana* (*C*. *meneghiniana*)	82
*Scenedesmus quadricauda* (*S*. *quadricauda*)	78
*Chroomonas acuta* (*C. acuta*)	74
*Navicula cryptocephala* (*N. cryptocephala*)	71
*Oscillatoria agardhii* (*O*. *agardhii*)	63
*Nitzschia palea* (*N*. *palea*)	59
*Chromulina* sp.	58
*Chlamydomonas globosa* (*C*. *globosa*)	57
*Synedra* sp.	53
*Cryptomonas ovata* (*C*.*ovata*)	52
*Gomphonema* sp.	45
*Nitzschia amphibia* (*N*.*amphibia*)	45
*Cocconeis placentula* (*C*. *placetula*)	39
*Oocystis lacustris* (*O*. *lacustris*)	38
*Navicula* sp.	35
*Pseudanabaena* sp.	34
*Euglena oxyuris* (*E*. *oxyuris*)	34
*Achnanthes* sp.	32
*Carteria* sp.	32
*Synedra ulna* (*S*. *ulna*)	32
*Synedra* sp.	31
*Nitzschia* sp.	30
*Cymbella tropica* (*C*. *tropica*)	28
*Aulacoseira granulata* (*A*. *granulata*)	28
*Phormidium* sp.	27
*Synedra acus* (*S*. *acus*)	26
*Melosira varians* (*M*. *varians*)	26
*Fragilaria* sp.	25
*Nitzschia reversa* (*N*. *reversa*)	22
*Ceratium hirundinella* (*C*. *hirundinella*)	20
*Closterium* sp.	17
*Euglena* sp.	15
*Mallomonas* sp.	9
*Euglena clavata* (*E*. *clavata*)	9
*Pandorina morum* (*P*. *morum*)	9
*Euglena* sp.	7
*Dinobryon* sp.	6
Dishui Lake (n = 70)	
*Chlamydomonas globosa* (*C*. *globosa*)	99
*Chromulina pygmaea* (*C*. *pygmaea*)	93
*Scenedesmus quadricauda* (*S*. *quadricauda*)	91
*Navicula* sp.	91
*Ankistrodesmus angustus* (*A*. *angustus*)	84
*Chaetoceros muelleri* (*C*. *muelleri*)	83
*Synedra acus* (*S*. *acus*)	83
*Oocystis lacustris* (*O*. *lacustris*)	81
*Chroomonas acuta* (*C*. *acuta*)	80
*Cocconeis placentula* (*C*. *palcentula*)	76
*Euglena* sp.	69
*Oscillatoria agardhii* (*O*. *agardhii*)	67
*Gymnodinium* sp.	66
*Cryptomonas erosa* (*C*. *erosa*)	63
*Ochromonas* sp.	59
*Cyclotella meneghiniana* (*C*. *meneghiniana*)	59
*Aulacoseira granulata* (*A*. *granulata*)	56
*Nitzschia* sp.	54
*Carteria* sp.	41
*Amphiprora* sp.	39
*Cryptomonas ovata* (*C*.*ovata*)	33
*Cyclotella* sp.	29
*Melosira varians* (*M*. *varians*)	29

### 3.1 The phytoplankton associations in HRB and Dishui Lake

Twelve phytoplankton associations in HRB were identified by combinations of two to three taxa (Table 5) based on support >= 50%, and confidence values of >=0.8. All associations can be divided into three types: (1) The flagellate algae association (R01‐1, R01‐2, R01‐9): pollution‐tolerant species (e.g., *Cryptomonas ovata* and *Cryptomonas erosa)* co‐existed; mixotrophic chrysophytes (e.g., *Chrumulina* sp.) was observed to occur with *Cryptomonas erosa*, *Chroomonas acuta*, *Chlamydomonas globosa* from different taxon groups. (2) The diatoms with the flagellate algae association (R03‐2, R05‐2, R06‐2, R07‐2, R08‐2, R10‐2, R11‐2, and R12‐2): benthic diatoms with motility (e.g., *Nitzschia palea*, *Navicula cryptocephala)* frequently co‐occurred with the cryptophytes. It should be noted that a small centric planktonic diatom (*Cyclotella meneghiniana*) also occurred with the flagellate algae. (3) The diatoms association (R04‐3: *Nitzschia palea* and *Cyclotella meneghiniana*).

**TABLE 5:** The phytoplankton associations composition in HRB and Dishui Lake (R:River;L:Lake)

Number + group	Taxa 1	Taxa 2	Taxa 3	Habitat template
R01‐1	*C*. *ovata*	*C*. *erosa*		Pollution, poor light, lentic
R02‐1	*Chromulina* sp.	*C*. *erosa*		Oligotrophic‐mesotrophic + pollution
R03‐2	*Chromulina* sp.	*C*. *meneghiniana*		Oligotrophic‐mesotrophic + silica‐rich, low P
R04‐3	*N*. *palea*	*C*. *meneghiniana*		Shallow turbid + silica‐rich, low P
R05‐2	*N*. *palea*	*C*. *erosa*		Shallow turbid + pollution
R06‐2	*N*. *cryptocephala*	*C*. *acuta*	*C*. *erosa*	Shallow turbid + pollution
R07‐2	*C*. *meneghiniana*	*N. cryptocephala*	*C*. *erosa*	Silica‐rich + shallow turbid + pollution
R08‐2	*C*. *acuta*	*C*. *globosa*	*C.meneghiniana*	Meso‐eutrophic + organic and inorganic nutrients
R09‐1	*C.acuta*	*C*. *globosa*	*C*. *erosa*	Meso‐eutrophic + pollution
R10‐2	*C*. *meneghiniana*	*C*. *acuta*	*C*. *erosa*	Silica‐rich, low P+ meso‐eutrophic
R11‐2	*C*. *meneghiniana*	*C*. *globosa*	*C*. *erosa*	Silica‐rich, low P+ meso‐eutrophic
R12‐2	*C*. *meneghiniana*	*O*. *agardhii*	*C*. *erosa*	Silica‐rich, low P+ meso‐eutrophic
L01‐2	*O*. *lacustris*	*C*. *globosa*		p‐limit + meso‐eutrophic
L02‐2	*S*. *acus*	*C*. *globosa*		Shallow turbid + meso‐eutrophic
L03‐2	*A*. *angustus*	*C*. *pygmaea*		Clear, mixed + shallow oligotrophic brackish
L04‐2	*A*. *angustus*	*C*. *globosa*		Clear, mixed + meso‐eutrophic
L05‐2	*C*. *muelleri*	*C*. *globosa*		Brackish, high NP+ meso‐eutrophic
L06‐3	*S*. *quadricauda*	*Navicula* sp.		Shallow, mixed + turbid shallow
L07‐2	*S*. *quadricauda*	*C*. *pygmaea*		Shallow mixed + shallow oligotrophic brackish
L08‐2	*S*. *quadricauda*	*C*. *globosa*		Shallow mixed + meso‐eutrophic
L09‐2	*Navicula* sp.	*C*. *pygmaea*		Turbid shallow + shallow oligotrophic brackish
L10‐2	*Navicula* sp.	*C*. *globosa*		Turbid shallow + meso‐eutrophic
L11‐1	*C*. *pygmaea*	*C*. *globosa*		Shallow oligotrophic brackish + meso‐eutrophic
L12‐2	*Navicula* sp.	*S*. *quadricauda*	*C*. *pygmaea*	Turbid + shallow mixed + shallow oligotrophic brackish
L13‐2	*Navicula* sp.	*S*. *quadricauda*	*C*. *globosa*	Turbid shallow + shallow mixed + meso‐eutrophic
L14‐2	*S*. *quadricauda*	*C*. *pygmaea*	*C*. *globosa*	Shallow + oligotrophic brackish + meso‐eutrophic
L15‐2	*Navicula* sp.	*C*. *pygmaea*	*C*. *globosa*	Turbid + shallow + oligotrophic brackish + meso‐eutrophic

Fifteen phytoplankton associations in Dishui Lake were identified by combinations of two to three taxa (Table 5). These associations can be divided into three types: (1) The flagellate algae association (L11‐1): mixotrophic chrysophyte *Chrumulina pygmaea* was observed to occur with *Chlamydomonas globosa*. (2) The mixed association‐diatoms or colonial green algae with the flagellate algae association: this association can be further divided into three smaller associations including mixotrophic chrysophyte *Chrumulina pygmaea* with diatoms or green algae (L03‐2, L07‐2, L09‐2, and L12‐2), *Chlamydomonas globosa* with diatoms or green algae (L01‐2, L02‐2, L04‐2, L05‐2, L08‐2, L10‐2, and L13‐2), and *Chromulina pygmaea* and *Chlamydomonas globosa* with diatoms or green algae (L14‐2 and L15‐2). (3) The diatoms with colonial green algae association (L06‐3).

### 3.2 Relationships between phytoplankton associations and environmental variables

We analyzed the phytoplankton assemblages characterized by 12 phytoplankton associations in HRB using detrended correspondence analysis (DCA). DCA results showed that the maximum gradient length of the four axes was 2.63. Subsequently, we selected a redundancy analysis (RDA) to detect the relationship between phytoplankton associations and environmental factors (Figure 2). Approximately, 12% of the variance in phytoplankton associations can be explained by environmental factors (axis 1: 8%, axis 2: 3%). Forward selection in RDA identified six significant environmental factors (Figure 2). Turbidity was positively correlated with axis 1, TN/TP ratio was negatively correlated with axis 1; Conductivity and TN positively correlated with axis 2, and stream order negatively correlated with axis 2. Most of the mixed associations had a positive relationship with TN and turbidity except that association 3 displayed a positive relationship with conductivity and a negative correlation with turbidity (Figure 2).The flagellate algae association had a positive relationship with turbidity, DO and stream size while the diatom association was positively associated with TN (Figure 2).

**FIGURE 3:**
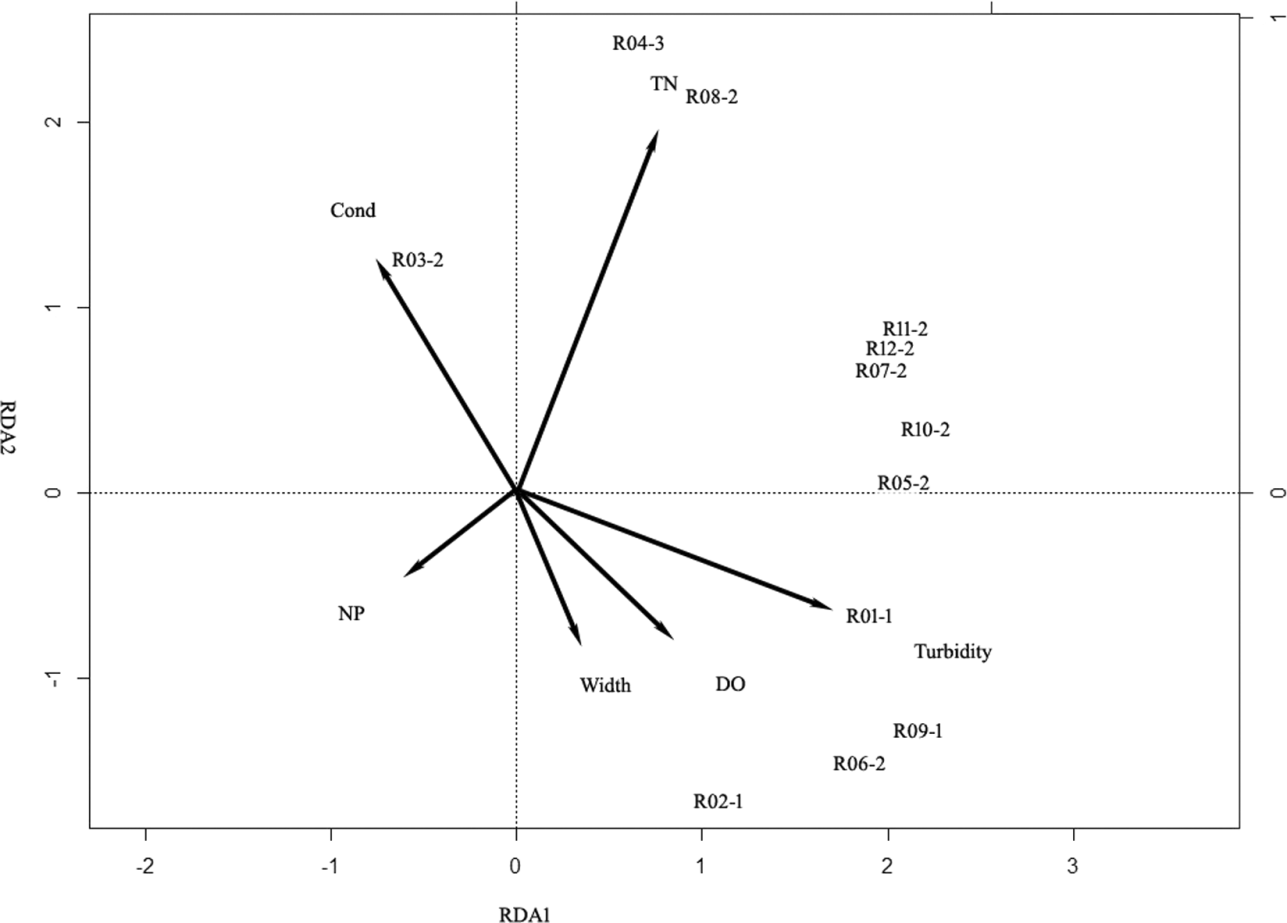
The RDA plot of 12 phytoplankton associations and environmental variables in HRB (DO: Dissolved oxygen; TN: Total nitrogen; NP:TN/TP ratio; Cond: Conductivity; phytoplankton association codes are in Table 5)

We used the 15 phytoplankton associations in Dishui Lake for detrended correspondence analysis (DCA) with the maximum gradient length of the four axes as 1.55. RDA showed that 32% variance in phytoplankton associations can be explained by environmental factors: axis 1: 27%, axis 2: 4.8%. Forward selection in RDA identified three significant environmental factors (Figure 3). Salinity and transparency were negatively correlated with axis 1; pH was negatively correlated with axis 2. The mixed associations including *Chromulina* had a positive relationship with pH and transparency while the mixed associations including *Chlamydomonas* had a negative relationship with pH (Figure 3). The flagellate associations positively correlated with salinity (Figure 3).

## 4 DISCUSSION

Compared to the traditional phytoplankton functional group development (Reynolds et al., 2002), affinity analysis encompasses a broad set of analytics techniques aimed at uncovering the connections of phytoplankton associations. Affinity analysis is a method for rapidly finding phytoplankton associations from a large data set. It has the advantage of time‐saving and easy use, especially for new algae researchers in a region with limited ecological studies on local phytoplankton assemblages. So affinity analysis can be used as a first step to identify candidate phytoplankton associations.

The identified phytoplankton associations reflect the ecological preferences of phytoplankton including the resource acquisition (e.g., light and nutrients) and competitive abilities (e.g., r/K selection or C‐S‐R model) (Salmaso et al., 2015). *Cryptomonas erosa* and *Cryptomonas ovata* or *Chroomonas acuta* from the same family were often concurrent in HRB (Table 2). These species can benefit from both mixotrophy and phagotrophy, and also can tolerate high dissolved nutrients and limiting light conditions (Graham & Wilcox, 2000; Kruk & Segura, 2012), and can avoid grazing by zooplankton. Some taxa from different divisions can form the associations such as diatom‐cryptophytes. Diatom‐cryptophytes associations are consistent with what Sommer et al. (1986) suggested that Cryptophyceae and small centric diatoms developed together when nutrients were available and light increased in spring because of their small volume and high growth rate. Some of the identified phytoplankton associations can reflect the grazing pressure. The observed associations are consistent with the notion that both *Scenedesmus* and *Selenastrum* are more resistant to grazing than either *Chlamydomonas* or *Ankistrodesmus*, while the latter two taxa are better competitors in the absence of grazing (Drake et al., 1993). Motile benthic diatoms such as *Nitzschia palea* are concurrent with some planktonic algae. Benthic diatom motility offers not only a selective advantage on silty substrata but also it is correlated with some ecological traits (Passy, 2007). Kawamura et al. (2004) demonstrated that the grazing pressure of gastropods had an influence on the *Nitzschia* species.

**FIGURE 4:**
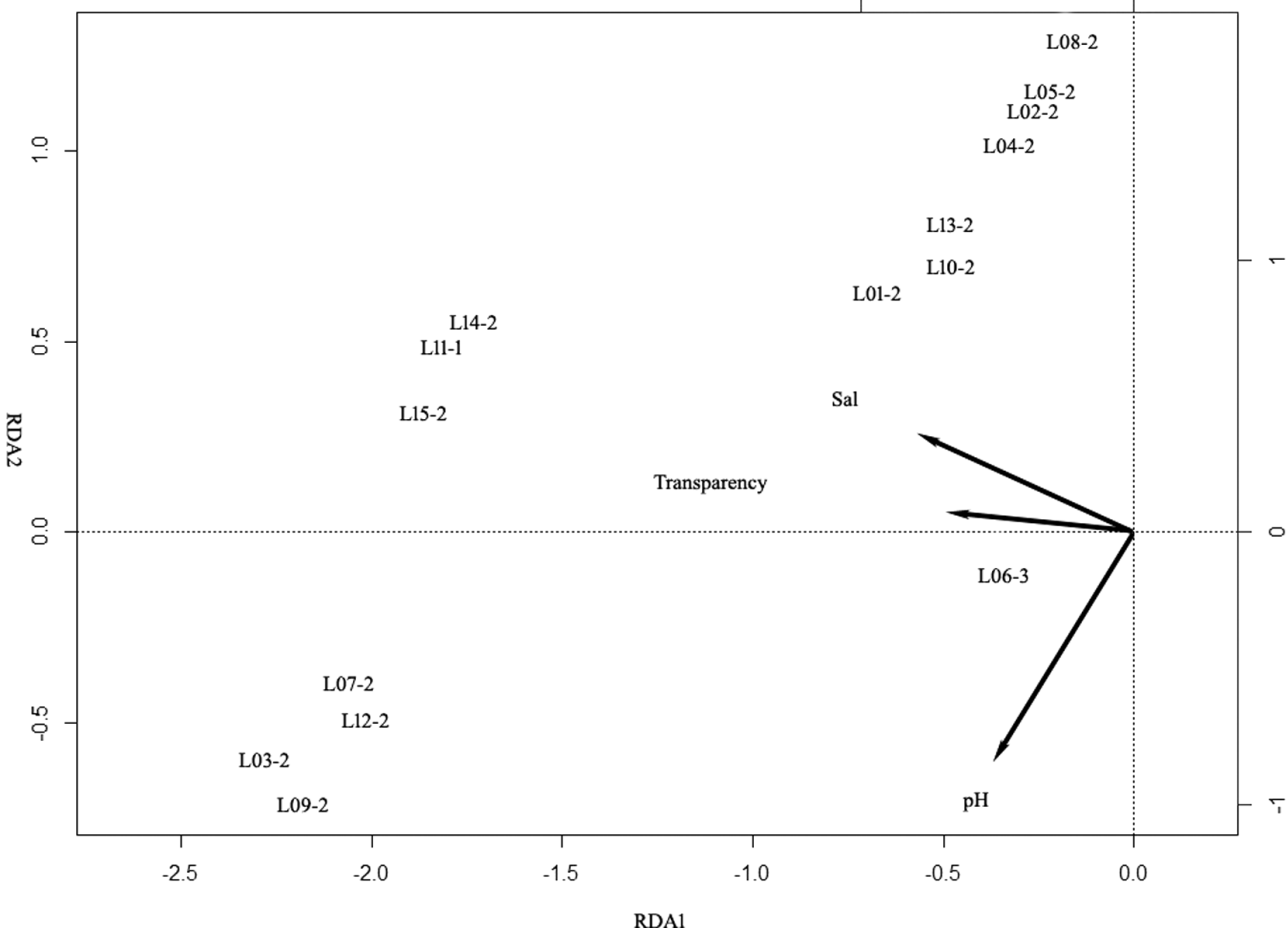
The RDA plot of 15 phytoplankton associations and environmental variables in Dishui Lake (Sal: Salinity; phytoplankton association codes are in Table 5)

We performed an RDA for assessing the applicability of the identified phytoplankton associations in environmental assessment. In HRB, light and TN were the best predictors of phytoplankton associations (Figure 3). Our results are consistent with (Mackay et al., 2012) that the diatom‐association was strong with the TN nutrient. In Dishui Lake, the light and salinity were the best predictors for phytoplankton associations (Figure 4). Chrysophytes are restricted to cold, oligotrophic conditions. Small *Chromulina* groups showed a different response to pH and water clarity, compared to the medium size *Chlamydomonas* groups (Figure 4). The importance of pH as a primary factor affecting chrysophytes has been reported in studies from widely separated geographic regions. *Chromulina* and *Chlamydomonas* are both *r*‐selected taxa, their small‐medium body size and motility conferred by flagella are advantages and allow them to reduce sinking rate (Kruk et al., 2010). Compared to the *Chlamydomonas*, the *Chromulina* prefer the oligotrophic environments with an abundance of macrophytes. Compared to the river, more variance (33%) in phytoplankton associations can be explained by different combinations of environmental factors in the man‐made shallow lake. A lake is perceived to be relatively stable, that of a river, and is characteristically graded from the origin to the river‐mouth (Reynolds et al., 1994). Therefore, candidate phytoplankton associations are reasonable proxies for explaining environmental variables.

Binary data were used to construct the phytoplankton associations in this work, which ignores the abundance of phytoplankton species. Although binary data are commonly observed and analyzed in many application fields (Yamamoto & Hayashi, 2015), some species which were not abundant potentially contributed much more to the analysis than those common taxa but we minimized the effects. The phytoplankton associations identified by affinity analysis should be viewed as candidate associations and each association should be carefully evaluated using ecological theories and concepts.

In essence, affinity analysis can be a useful method for finding the phytoplankton associations from the complex and informative data set. It can explain some fraction of the variance from both spatial and temporal algal assemblages distribution patterns, although their effectiveness varies differently in rivers and lakes, depending on the gradients of environmental factors. Our results do not mean that the proposed method should replace the conventional ecological classifications of phytoplankton. The proposed method provides an alternative, especially for the regions where researches on phytoplankton assemblages are still limited. Affinity analysis remains to be tested in future research on whether it is predicted better for phytoplankton associations than other classification systems.

## AUTHOR CONTRIBUTIONS

**Weiju Zhu:** Writing – Performed the data analyses and lead the writing. **Zhaojian Ding:** Writing – review and editing (supporting). **Yangdong Pan:** Conceived the idea. **Quanxi Wang:** Performed the data collection.

## CONFLICT OF INTEREST

The authors declared that they have no conflicts of interest to this work. We declare that we do not have any commercial or associative interest that represents a conflict of interest in connection with the work submitted.

## Data Availability

The data that support the findings of this study are available online: 4TU Research Data. https://doi.org/10.4121/18487790.
